# Methyl 5-bromo-2-chloro­pyridine-3-carboxyl­ate

**DOI:** 10.1107/S1600536808013305

**Published:** 2008-05-14

**Authors:** Yi Ma, Ya-Lun Liu

**Affiliations:** a School of Chemical Engineering, Shandong Institute of Light Industry, Jinan 250353, People’s Republic of China; b Jinan China Cotton Industry Co. Ltd, Jinan 250100, People’s Republic of China

## Abstract

The title compound, C_7_H_5_BrClNO_2_, crystallizes with two independent molecules in the asymmetric unit. In the absence of classical inter­molecular inter­actions, the crystal structure exhibits relatively short inter­molecular Br⋯O distances [3.143 (9) and 3.162 (9)Å].

## Related literature

For the biological activity of the title compound, see: Colarusso & Narjes (2004 ▶); Kim *et al.* (2006 ▶). For related crystal structures, see McArdle *et al.* (1982 ▶).
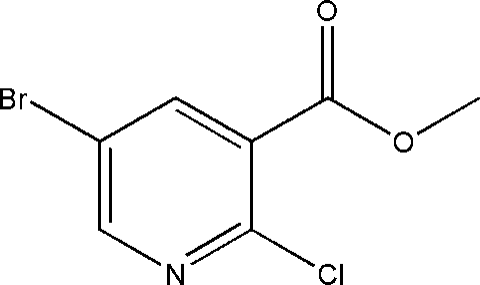


## Experimental

### 

#### Crystal data

C_7_H_5_BrClNO_2_
*M* *_r_* = 250.48
Triclinic, 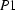

*a* = 3.978 (2) Å
*b* = 8.153 (3) Å
*c* = 14.040 (2) Å
α = 96.89 (2)°
β = 96.20 (3)°
γ = 100.70 (2)°
*V* = 440.2 (3) Å^3^
*Z* = 2
Mo *K*α radiation
μ = 4.93 mm^−1^
*T* = 298 (2) K
0.16 × 0.14 × 0.10 mm

#### Data collection

Bruker SMART CCD area-detector diffractometer
Absorption correction: multi-scan (*SADABS*; Sheldrick, 2004 ▶) *T*_min_ = 0.506, *T*_max_ = 0.638
2186 measured reflections
1818 independent reflections
1564 reflections with *I* > 2σ(*I*)
*R*_int_ = 0.017

#### Refinement

*R*[*F* ^2^ > 2σ(*F* ^2^)] = 0.044
*wR*(*F* ^2^) = 0.130
*S* = 1.09
1818 reflections
217 parameters
3 restraints
H-atom parameters constrained
Δρ_max_ = 1.17 e Å^−3^
Δρ_min_ = −0.90 e Å^−3^
Absolute structure: Flack (1983 ▶); 70 Friedel pairs
Flack parameter: 0.01 (2)

### 

Data collection: *SMART* (Bruker, 2001 ▶); cell refinement: *SAINT* (Bruker, 2001 ▶); data reduction: *SAINT*; program(s) used to solve structure: *SHELXTL* (Sheldrick, 2008 ▶); program(s) used to refine structure: *SHELXTL*; molecular graphics: *SHELXTL*; software used to prepare material for publication: *SHELXTL* and local programs.

## Supplementary Material

Crystal structure: contains datablocks I, global. DOI: 10.1107/S1600536808013305/cv2402sup1.cif

Structure factors: contains datablocks I. DOI: 10.1107/S1600536808013305/cv2402Isup2.hkl

Additional supplementary materials:  crystallographic information; 3D view; checkCIF report

## Figures and Tables

**Table 1:** Selected interatomic distances (Å)

Br1⋯O3^i^	3.143 (9)
Br2⋯O1^ii^	3.162 (9)

Symmetry codes: (i) 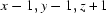; (ii) 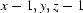.

**Fig. 1.**
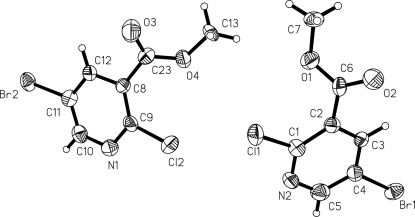
Two independent molecules of (I) with atomic numbering and displacement ellipsoids drawn at the 40% probability level.

## supplementary crystallographic information

### Comment

The title compound, (I), is a useful intermediate for the synthesis of various bioactive compounds (Colarusso *et al.*, 2004; Kim *et al.*, 2006). In this paper, we report its crystal structure.

Compound (I) crystallizes with two independent molecules in the non-centrosymmetric triclinic unit cell (Fig. 1). The bond lengths and angles in the molecules are normal and in a good agreement with those reported previously (McArdle *et al.*, 1982). The dihedral angles between the planes of the methoxycarbonyl group (C6/C7/O1/O2; C13/C23/O3/O4) and pyridine rings in the two independent molecules are 45.8 (2) and 44.0 (3)°, respectively. In the abscence of classical intermolecular interactions, the crystal packing exhibits relatively short intermolecular Br···O distances (Table 1).

### Experimental

A solution of 5-bromo-2-hydroxynicotinic acid (0.138 mol) and N, *N*-dimethylformamide (0.138 mol) in thionyl chloride (160 mL) was refluxed for 2 h. Thionyl chloride was evaporated and the yellow residue dissolved in anhydrous dichloromethane (200 mL), then anhydrous methanol was added dropwise. The resulting mixture was refluxed for 1 h and evaporated to afford slightly yellow oil which crystallized upon standing at room temperature. Crystals suitable for X-ray diffraction analysis were obtained by slow evaporation of an ethanol solution at room temperature over a period of one week.

### Refinement

All H atoms were placed in calculated positions, with C—H = 0.93 or 0.96 Å, and included in the final cycles of refinement using a riding model, with *U*_iso_(H) = 1.2–1.5 times *U*_eq_(C).

### Crystal data

**Table:**

C_7_H_5_BrClNO_2_	*Z* = 2
*M**_r_* = 250.48	*F*_000_ = 244
Triclinic, *P*1	*D*_x_ = 1.890 Mg m^−^^3^
Hall symbol: P 1	Mo *K*α radiation λ = 0.71073 Å
*a* = 3.978 (2) Å	Cell parameters from 947 reflections
*b* = 8.153 (3) Å	θ = 2.6–24.3º
*c* = 14.040 (2) Å	µ = 4.93 mm^−^^1^
α = 96.89 (2)º	*T* = 298 (2) K
β = 96.20 (3)º	Block, colourless
γ = 100.70 (2)º	0.16 × 0.14 × 0.10 mm
*V* = 440.2 (3) Å^3^	

### Data collection

**Table:**

Bruker SMART CCD area-detector diffractometer	1818 independent reflections
Radiation source: fine-focus sealed tube	1564 reflections with *I* > 2σ(*I*)
Monochromator: graphite	*R*_int_ = 0.017
*T* = 298(2) K	θ_max_ = 25.0º
φ and ω scans	θ_min_ = 1.5º
Absorption correction: multi-scan(SADABS; Sheldrick, 2004)	*h* = −4→3
*T*_min_ = 0.506, *T*_max_ = 0.639	*k* = −9→9
2186 measured reflections	*l* = −14→16

### Refinement

**Table:**

Refinement on *F*^2^	Hydrogen site location: inferred from neighbouring sites
Least-squares matrix: full	H-atom parameters constrained
*R*[*F*^2^ > 2σ(*F*^2^)] = 0.044	*w* = 1/[σ^2^(*F*_o_^2^) + (0.0806*P*)^2^] where *P* = (*F*_o_^2^ + 2*F*_c_^2^)/3
*wR*(*F*^2^) = 0.130	(Δ/σ)_max_ < 0.001
*S* = 1.09	Δρ_max_ = 1.17 e Å^−^^3^
1818 reflections	Δρ_min_ = −0.90 e Å^−^^3^
217 parameters	Extinction correction: none
3 restraints	Absolute structure: Flack (1983); 70 Friedel pairs
Primary atom site location: structure-invariant direct methods	Flack parameter: 0.01 (2)
Secondary atom site location: difference Fourier map	

### Special details

**Table:**

Geometry. All e.s.d.'s (except the e.s.d. in the dihedral angle between two l.s. planes) are estimated using the full covariance matrix. The cell e.s.d.'s are taken into account individually in the estimation of e.s.d.'s in distances, angles and torsion angles; correlations between e.s.d.'s in cell parameters are only used when they are defined by crystal symmetry. An approximate (isotropic) treatment of cell e.s.d.'s is used for estimating e.s.d.'s involving l.s. planes.
Refinement. Refinement of *F*^2^ against ALL reflections. The weighted *R*-factor *wR* and goodness of fit *S* are based on *F*^2^, conventional *R*-factors *R* are based on *F*, with *F* set to zero for negative *F*^2^. The threshold expression of *F*^2^ > σ(*F*^2^) is used only for calculating *R*-factors(gt) *etc*. and is not relevant to the choice of reflections for refinement. *R*-factors based on *F*^2^ are statistically about twice as large as those based on *F*, and *R*- factors based on ALL data will be even larger.

### Fractional atomic coordinates and isotropic or equivalent isotropic displacement parameters (Å^2^)

**Table:**

	*x*	*y*	*z*	*U*_iso_*/*U*_eq_	
Br1	0.3498 (2)	0.81123 (11)	0.74398 (8)	0.0523 (3)	
Br2	0.3533 (3)	1.31291 (12)	−0.24715 (8)	0.0530 (3)	
Cl1	0.8539 (9)	0.7931 (4)	0.3331 (2)	0.0552 (7)	
Cl2	1.2578 (8)	1.5050 (4)	0.1624 (2)	0.0555 (7)	
O1	1.083 (3)	1.2390 (10)	0.5287 (6)	0.074 (3)	
O2	0.762 (2)	1.1535 (9)	0.3865 (5)	0.0522 (19)	
O3	1.295 (3)	1.8504 (10)	−0.0331 (6)	0.080 (3)	
O4	1.116 (2)	1.8360 (9)	0.1109 (5)	0.0529 (19)	
N1	0.579 (3)	0.6646 (10)	0.4701 (7)	0.050 (2)	
N2	0.843 (3)	1.3036 (11)	0.0259 (7)	0.053 (2)	
C1	0.703 (3)	0.8099 (12)	0.4449 (7)	0.040 (2)	
C2	0.735 (3)	0.9674 (12)	0.5010 (7)	0.037 (2)	
C3	0.627 (3)	0.9656 (12)	0.5894 (7)	0.038 (2)	
H3A	0.6454	1.0663	0.6303	0.045*	
C4	0.490 (3)	0.8134 (12)	0.6187 (7)	0.042 (2)	
C5	0.464 (3)	0.6665 (14)	0.5574 (8)	0.055 (3)	
H5A	0.3658	0.5650	0.5764	0.066*	
C6	0.880 (3)	1.1312 (12)	0.4727 (7)	0.043 (2)	
C7	0.885 (3)	1.3097 (13)	0.3515 (8)	0.050 (3)	
H7A	0.7729	1.3062	0.2869	0.076*	
H7B	1.1297	1.3255	0.3511	0.076*	
H7C	0.8330	1.4015	0.3931	0.076*	
C8	0.995 (3)	1.4644 (13)	0.0517 (8)	0.042 (2)	
C9	0.973 (3)	1.5908 (12)	−0.0031 (7)	0.039 (2)	
C10	0.783 (3)	1.5431 (12)	−0.0957 (7)	0.042 (2)	
H10A	0.7706	1.6217	−0.1381	0.050*	
C11	0.616 (3)	1.3777 (12)	−0.1223 (7)	0.040 (2)	
C12	0.647 (3)	1.2631 (13)	−0.0611 (8)	0.049 (3)	
H12A	0.5287	1.1523	−0.0800	0.059*	
C13	1.144 (3)	1.7723 (13)	0.0234 (7)	0.042 (2)	
C14	1.289 (3)	2.0069 (11)	0.1478 (8)	0.047 (3)	
H14A	1.2448	2.0350	0.2130	0.071*	
H14B	1.2055	2.0824	0.1079	0.071*	
H14C	1.5334	2.0172	0.1470	0.071*	

### Atomic displacement parameters (Å^2^)

**Table:**

	*U*^11^	*U*^22^	*U*^33^	*U*^12^	*U*^13^	*U*^23^
Br1	0.0550 (6)	0.0621 (6)	0.0479 (7)	0.0190 (5)	0.0166 (5)	0.0206 (5)
Br2	0.0487 (6)	0.0544 (6)	0.0496 (7)	0.0118 (4)	−0.0056 (5)	−0.0094 (5)
Cl1	0.068 (2)	0.0575 (15)	0.0389 (14)	0.0144 (14)	0.0110 (12)	−0.0028 (12)
Cl2	0.067 (2)	0.0589 (16)	0.0397 (14)	0.0138 (14)	−0.0012 (12)	0.0113 (12)
O1	0.092 (7)	0.049 (4)	0.064 (5)	−0.012 (5)	−0.013 (5)	0.010 (4)
O2	0.058 (5)	0.050 (4)	0.047 (4)	0.005 (4)	0.004 (4)	0.013 (3)
O3	0.115 (8)	0.056 (5)	0.059 (5)	−0.019 (5)	0.029 (5)	0.005 (4)
O4	0.073 (6)	0.041 (4)	0.042 (4)	0.005 (4)	0.014 (4)	−0.001 (3)
N1	0.059 (7)	0.034 (5)	0.053 (6)	0.009 (4)	−0.006 (5)	−0.001 (4)
N2	0.066 (7)	0.044 (5)	0.048 (6)	0.007 (5)	0.012 (5)	0.011 (4)
C1	0.035 (6)	0.041 (6)	0.043 (6)	0.006 (4)	0.007 (5)	0.003 (5)
C2	0.033 (6)	0.037 (5)	0.040 (5)	0.005 (4)	−0.002 (4)	0.006 (4)
C3	0.042 (6)	0.041 (5)	0.031 (5)	0.011 (4)	0.007 (4)	0.006 (4)
C4	0.039 (6)	0.045 (6)	0.043 (6)	0.013 (4)	0.002 (4)	0.004 (4)
C5	0.057 (8)	0.047 (6)	0.057 (7)	0.005 (5)	−0.001 (5)	0.010 (5)
C6	0.043 (7)	0.044 (6)	0.035 (5)	0.005 (5)	−0.001 (4)	−0.001 (4)
C7	0.070 (8)	0.037 (6)	0.049 (7)	0.005 (5)	0.024 (6)	0.019 (5)
C8	0.049 (7)	0.039 (6)	0.041 (6)	0.015 (5)	0.015 (5)	0.002 (4)
C9	0.047 (6)	0.038 (5)	0.033 (5)	0.006 (5)	0.013 (4)	0.006 (4)
C10	0.051 (7)	0.039 (5)	0.037 (5)	0.011 (5)	0.012 (4)	0.005 (4)
C11	0.031 (6)	0.045 (6)	0.044 (6)	0.004 (4)	0.004 (4)	0.005 (4)
C12	0.049 (7)	0.042 (6)	0.052 (6)	0.003 (5)	0.009 (5)	−0.001 (5)
C13	0.038 (6)	0.049 (6)	0.039 (6)	0.007 (5)	0.006 (4)	0.011 (4)
C14	0.060 (7)	0.024 (5)	0.051 (7)	0.003 (5)	0.000 (5)	−0.004 (4)

### Geometric parameters (Å, °)

**Table:**

Br1—C4	1.902 (11)	C3—C4	1.389 (14)
Br2—C11	1.902 (10)	C3—H3A	0.9300
Cl1—C1	1.740 (10)	C4—C5	1.370 (15)
Cl2—C8	1.736 (12)	C5—H5A	0.9300
O1—C6	1.215 (11)	C7—H7A	0.9600
O2—C6	1.296 (11)	C7—H7B	0.9600
O2—C7	1.439 (12)	C7—H7C	0.9600
O3—C13	1.215 (11)	C8—C9	1.368 (14)
O4—C13	1.299 (13)	C9—C10	1.403 (13)
O4—C14	1.441 (12)	C9—C13	1.494 (14)
N1—C1	1.302 (12)	C10—C11	1.376 (13)
N1—C5	1.351 (15)	C10—H10A	0.9300
N2—C8	1.327 (13)	C11—C12	1.357 (13)
N2—C12	1.346 (14)	C12—H12A	0.9300
C1—C2	1.400 (13)	C14—H14A	0.9600
C2—C3	1.357 (13)	C14—H14B	0.9600
C2—C6	1.471 (13)	C14—H14C	0.9600
			
Br1···O3^i^	3.143 (9)	Br2···O1^ii^	3.162 (9)
			
C6—O2—C7	119.8 (8)	H7A—C7—H7C	109.5
C13—O4—C14	119.5 (9)	H7B—C7—H7C	109.5
C1—N1—C5	117.0 (9)	N2—C8—C9	125.6 (11)
C8—N2—C12	116.7 (9)	N2—C8—Cl2	114.0 (8)
N1—C1—C2	125.8 (9)	C9—C8—Cl2	120.4 (8)
N1—C1—Cl1	113.3 (8)	C8—C9—C10	116.4 (9)
C2—C1—Cl1	120.9 (7)	C8—C9—C13	126.8 (9)
C3—C2—C1	116.1 (8)	C10—C9—C13	116.7 (8)
C3—C2—C6	118.3 (9)	C11—C10—C9	118.6 (8)
C1—C2—C6	125.6 (9)	C11—C10—H10A	120.7
C2—C3—C4	120.0 (9)	C9—C10—H10A	120.7
C2—C3—H3A	120.0	C12—C11—C10	120.1 (9)
C4—C3—H3A	120.0	C12—C11—Br2	121.1 (7)
C5—C4—C3	119.2 (10)	C10—C11—Br2	118.8 (7)
C5—C4—Br1	121.0 (8)	N2—C12—C11	122.5 (9)
C3—C4—Br1	119.8 (7)	N2—C12—H12A	118.8
N1—C5—C4	122.0 (10)	C11—C12—H12A	118.8
N1—C5—H5A	119.0	O3—C13—O4	124.2 (9)
C4—C5—H5A	119.0	O3—C13—C9	121.9 (9)
O1—C6—O2	123.3 (9)	O4—C13—C9	114.0 (8)
O1—C6—C2	121.6 (9)	O4—C14—H14A	109.5
O2—C6—C2	115.0 (8)	O4—C14—H14B	109.5
O2—C7—H7A	109.5	H14A—C14—H14B	109.5
O2—C7—H7B	109.5	O4—C14—H14C	109.5
H7A—C7—H7B	109.5	H14A—C14—H14C	109.5
O2—C7—H7C	109.5	H14B—C14—H14C	109.5
			
C5—N1—C1—C2	1.3 (16)	C12—N2—C8—C9	−0.8 (17)
C5—N1—C1—Cl1	178.3 (8)	C12—N2—C8—Cl2	−177.4 (8)
N1—C1—C2—C3	0.5 (16)	N2—C8—C9—C10	−2.7 (16)
Cl1—C1—C2—C3	−176.3 (7)	Cl2—C8—C9—C10	173.7 (8)
N1—C1—C2—C6	179.1 (9)	N2—C8—C9—C13	−179.3 (11)
Cl1—C1—C2—C6	2.4 (15)	Cl2—C8—C9—C13	−3.0 (15)
C1—C2—C3—C4	−1.0 (14)	C8—C9—C10—C11	4.3 (14)
C6—C2—C3—C4	−179.8 (10)	C13—C9—C10—C11	−178.7 (9)
C2—C3—C4—C5	−0.1 (15)	C9—C10—C11—C12	−2.6 (15)
C2—C3—C4—Br1	178.4 (8)	C9—C10—C11—Br2	179.2 (8)
C1—N1—C5—C4	−2.5 (16)	C8—N2—C12—C11	2.8 (16)
C3—C4—C5—N1	2.0 (16)	C10—C11—C12—N2	−1.1 (16)
Br1—C4—C5—N1	−176.5 (8)	Br2—C11—C12—N2	177.1 (9)
C7—O2—C6—O1	2.9 (18)	C14—O4—C13—O3	−3.9 (17)
C7—O2—C6—C2	179.6 (10)	C14—O4—C13—C9	175.8 (10)
C3—C2—C6—O1	43.6 (16)	C8—C9—C13—O3	133.7 (12)
C1—C2—C6—O1	−135.1 (12)	C10—C9—C13—O3	−42.9 (15)
C3—C2—C6—O2	−133.2 (10)	C8—C9—C13—O4	−46.0 (15)
C1—C2—C6—O2	48.2 (15)	C10—C9—C13—O4	137.4 (10)

Symmetry codes: (i) *x*−1, *y*−1, *z*+1; (ii) *x*−1, *y*, *z*−1.
